# Pupillenrekonstruktion mit einer künstlichen Iris

**DOI:** 10.1007/s00347-021-01406-4

**Published:** 2021-06-28

**Authors:** Christian Mayer, Ramin Khoramnia

**Affiliations:** Universitätsaugenklinik Heidelberg, Ruprecht-Karls-Universität Heidelberg, Im Neuenheimer Feld 400, 69120 Heidelberg, Deutschland

**Keywords:** Okuläres Trauma, Aniridie, Aphakie, IOL, Blendung, Ocular trauma, Aniridia, Aphakia, IOL, Glare

## Abstract

**Hintergrund:**

Patienten mit Irisdefekten leiden unter starken Sehbeeinträchtigungen, insbesondere erhöhter Blendungsempfindlichkeit, und kosmetischen Beeinträchtigungen. Dies kann eine große psychische Belastung für die betroffenen Patienten darstellen. In der jüngeren Vergangenheit bestand die Behandlung aus farbigen Iriskontaktlinsen, Sonnenbrillen und einfachen Irisprothesen. Indikationen für eine chirurgische Behandlung sind Kolobome der Iris, Aniridie, traumatische Irisdefekte und persistierende Mydriasis. Ziel dieser Übersichtsarbeit ist es, chirurgische Ansätze, funktionelle und ästhetische Ergebnisse sowie Komplikationen nach Implantation einer individuellen künstlichen Irisprothese aus Silikon zu untersuchen.

**Ziel der Arbeit:**

Analyse der veröffentlichten Literatur zum Thema chirurgische Irisrekonstruktion mit einer künstlichen Iris in Kombination mit eigenen Erfahrungen über 120 vom Autor in den letzten 10 Jahren behandelter Patienten.

**Material und Methoden:**

Die in dieser Übersichtsarbeit verwendete maßgefertigte, flexible Silikonirisprothese Artificial*Iris* (HumanOptics, Erlangen, Deutschland) ist eine innovative und vielseitige Option in der chirurgischen Behandlung von Irisdefekten. Besprochen werden die diversen Implantationstechniken, die erreichbaren Ergebnisse sowie die möglichen Komplikationen.

**Ergebnisse:**

Untersucht wurden die Veränderung der bestkorrigierten Sehschärfe, der Augeninnendruck, die Pupillenöffnung, die Blendung, die Kontrastempfindlichkeit, die Endothelzellzahl, die Vorderkammertiefe, der Kammerwinkel und die Patientenzufriedenheit. Weiterhin wurden Komplikationen und die Farbanpassung an die Rest- und Partneraugeniris bewertet.

**Diskussion:**

Die Implantation der künstlichen Iris ist eine effektive Therapieoption zur Behandlung ausgeprägter traumatischer Irisdefekte und führt neben einer hohen Patientenzufriedenheit zu einem individuellen, ästhetisch ansprechenden und guten funktionellen Ergebnis. Es handelt sich aber um einen nicht zu unterschätzenden Eingriff mit flacher Lernkurve, bei dem Komplikationen auftreten können.

## Abstract (en)

**Background:**

Patients with iris defects suffer from severe visual impairment, especially increased glare sensitivity, and cosmetic disturbances. This can constitute a great psychological strain for patients. In the recent past the treatment consisted of iris print contact lenses, sunglasses and simple iris prostheses. The indications for surgical treatment are colobomas of the iris, aniridia, traumatic iris defects and persistent mydriasis. The aim of this review article is to investigate the surgical approaches, complications, functional and aesthetic outcome after implantation of an individual artificial iris prosthesis made from silicone.

**Objective:**

Analysis of the literature on the topic of surgical iris reconstruction with an artificial iris in combination with the own experience in more than 120 patients treated by the author in the last 10 years.

**Material and methods:**

The custom-made flexible silicone iris prosthesis Artificial*Iris* (HumanOptics, Erlangen, Germany) assessed in this review is an innovative and versatile option for surgical treatment of iris defects. Patients were examined before and after iris reconstruction with respect to feasibility, complications and outcome.

**Results:**

Change of best corrected visual acuity, intraocular pressure, pupillary opening, glare, contrast sensitivity, endothelial cell count, anterior chamber depth, anterior chamber angle and patient satisfaction were assessed. Furthermore, complications and color match to the residual and fellow iris were assessed.

**Conclusion:**

The implantation of an artificial iris is an effective option for the treatment of extensive traumatic iris defects and leads to an individual aesthetically appealing and good functional outcome as well as high patient satisfaction; however, this is an intervention that should not be underestimated because of a flat learning curve and various complications that can occur.

Irisdefekte sind meist Folgeschäden nach stumpfen oder perforierenden okulären Traumata. Das Ausmaß eines Irisdefektes reicht von einer persistierenden traumatischen Mydriasis über Iristeilverluste bis hin zur kompletten Aniridie. Neben Blendung, verminderter Schärfentiefe und vermindertem Kontrastsehen leiden die Betroffenen meist noch an einer ästhetischen Beeinträchtigung (Abb. 1a). Hervorzuheben ist, dass oftmals die geschädigten Augen neben den Irisdefekten weitere, zum Teil schwerwiegende Verletzungsfolgen wie Glaukom, Aphakie, Refraktionsfehler sowie Hornhaut- oder Netzhautnarben aufweisen. Bei alleiniger Pupillenrekonstruktion ist eine Visusverbesserung nicht primäres Therapieziel. Einige Patienten, die z. B. an einer kongenitalen Aniridie oder bilateralen Iriserkrankungen leiden, bedürfen sogar einer beidseitigen Irisrekonstruktion [12].

**Figure 1:**
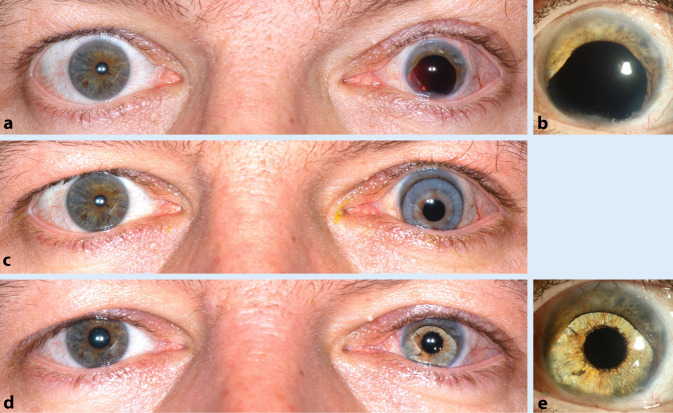

## Konservative Therapieoptionen

### Licht(blend)schutzbrillen

Diese bieten verschiedene Tönungsgrade und können mit Seitenschutz und Schirmmütze kombiniert werden. Ein Trageversuch ist in jedem Fall zu empfehlen. Sie stellen eine erste oder dauerhafte praktikable und reversible Maßnahme dar.

### Bedruckte Iriskontaktlinsen

Sie reduzieren durch eine aufgedruckte Iris mit kleinem Pupillendurchmesser die in das Auge einfallende Lichtmenge (Abb. 1c und 2a). Ebenso können die Kontaktlinsen gleichzeitig Refraktionsanomalien ausgleichen. Nachteil bei Kontaktlinsen sind aber die Notwendigkeit einer konsequenten und zeitaufwendigen Pflege, die hohen laufenden Kosten und evtl. auch die Gefahr einer Trageunverträglichkeit. Iriskontaktlinsen sollten im Falle einer geplanten operativen Maßnahme im Rahmen eines präoperativen Trageversuchs dafür genutzt werden, den Patienten vor dem Eingriff das potenziell erreichbare Ergebnis zu demonstrieren [13].

**Figure 2:**
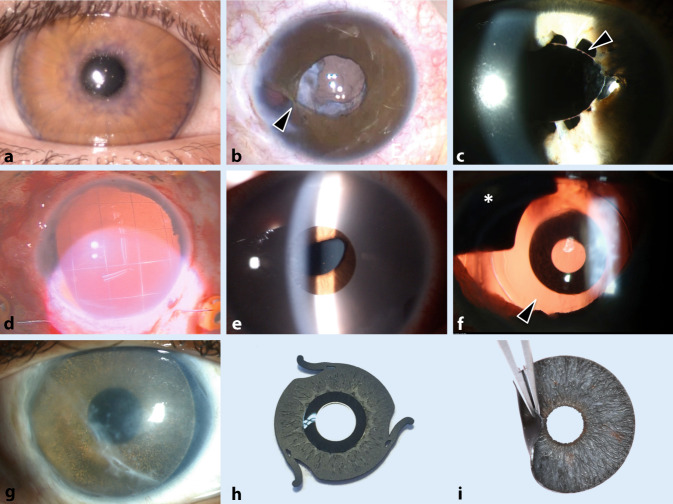

## Operative Therapieoptionen

Es stehen prinzipiell verschiedene pupillenrekonstruktive Maßnahmen ohne und mit Irisprothesen zur Verfügung ([10, 30, 34, 35]; Abb. 2b–i). Vielen betroffenen Patienten und deren betreuenden Augenärzten sind die verschiedenen Versorgungsalternativen nicht alle bekannt.

### Irisnähte

Kleine sektorielle Irisdefekte bis ca. 2 Uhrzeiten, Irisbasisdefekte sowie umschriebene Löcher können meistens gut mit Irisnähten versorgt werden (Abb. 2b, c; [4]).

Vorteilhaft bei Nähten ist, dass vorhandene Irisbestandteile verwendet und keine intraokularen Implantate benötigt werden. Bei richtiger Indikation lassen sich gute ästhetische und funktionelle Ergebnisse erzielen. Nachteilig sind die längere Operationsdauer besonders bei aufwendigen und komplexen intrakameralen Nahttechniken und die Möglichkeit, nur kleinere Defekte behandeln zu können. Es kann anspruchsvoll sein, lichtdichte Gewebeadaptationen zu schaffen.

Im Rahmen einer Primärversorgungssituation kann die Verwendung von Silikonölretentionsnähten („Iris-Gitternaht“; Abb. 2d) bei aphaken und aniriden Augen verhindern, dass Silikonöl in die Vorderkammer gelangt und mit dem Hornhautendothel in Kontakt kommt. In einem geplanten Folgeeingriff kann dann ein sekundäres Iris-Linsen-Diaphragma implantiert werden [18].

### Irisprothesen für die Vorderkammer

Die weder in Deutschland noch in den USA zugelassenen Irisprothesen NewColorIris (Kahn Medical Devices, Corp) und BrightOcular (Stellar Devices LLC) werden mit mehreren Farbvarianten vermarktet (Abb. 2e). Die Silikoniris ist faltbar und wird stets in die Vorderkammer *vor* eine bestehende Iris implantiert, um die Augenfarbe zu ändern: In nahezu allen Fällen kommt es zu einem Glaukom, einer Endotheldekompensation und einer chronischen Iritis [6, 8, 27]. Aus Sicht der Autoren darf diese künstliche Iris aufgrund des schlechten Sicherheitsprofils nicht zur Implantation eingesetzt werden und muss – sofern bereits implantiert – zeitnah wieder entfernt werden.

### Irissegmentimplantate

Irissegmentringe (z. B. von der Firma Morcher) in schwarz ohne Optik können zusammen mit einer Intraokularlinse (IOL) in den Kapselsack implantiert werden und so rotiert werden, dass der Irisdefekt von der Prothese bedeckt wird (Abb. 2f, Sternchen). Darüber hinaus können die Segmente auch im Sulcus ciliaris fixiert bzw. eingenäht werden. Sie bestehen aus einem starren Material und erfordern meist eine relativ große Inzision zur Implantation. Da diese Implantate aus einem schwarzen Material bestehen, sind die ästhetischen Ergebnisse (v. a. bei heller Irisfarbe) nicht immer optimal.

### Volliris-(IOL-)Implantate

Derzeitig vermarktete alternative Therapieoptionen (Tab. 1) sind z. T. kombinierte Irisblenden-IOL-Implantate:

- Die Gruppe der Aniridieimplantate von Morcher (Stuttgart, Deutschland) unterscheiden sich in Form, Größe und optischem Einsatz. Die Implantate sind aus nicht flexiblem Poly-Methyl-Methacrylat (PMMA) und stets in schwarzer Farbe gefertigt [24].
- Das Implantat von Ophtec (Emmerich, Deutschland) bietet Farbvielfalt, um dem verbliebenen Irisgewebe oder der Iris des Partnerauges zu entsprechen, und 2 verschiedene Haptikdesigns mit oder ohne optischer Linse (Abb. 2h; [7]).
- Die Silikonirisblende zur (vorübergehenden) Verwendung mit Silikonöl MICROSIL® DIAPHRAGM DP 4128 (HumanOptics, Erlangen, Deutschland). Aufgrund der weiß-transparenten Farbe und der fehlenden brechenden Wirkung erfüllt diese Blende keinen ästhetischen und refraktiven Anspruch. Sie dient vielmehr als provisorische Option im Rahmen einer notfallmäßig durchzuführenden Operation.
- Die Customflex Artificial*Iris* (AI; HumanOptics) (Abb. 2i). Aufgrund der intensiven Erfahrungen mit dieser Irisprothese wird ihre Anwendung im Folgenden näher betrachtet.

**Table 1:** 

Modell	Optik	Faltbar	Farbauswahl	Material	Preis (brutto)
Microsil Diaphragm (HumanOptics)	Planes Kunststofffenster	Ja	Weiß	Silikon	ca. 963,00 €/Paar
Customflex Artificial*Iris* (HumanOptics)	Nein	Ja	Individuell angefertigt	Silikon	ca. 3156,50 €
Aniridieimplantat u. a. Type 67 oder 68 (Morcher)	Ja	Nein	Schwarz	PMMA	ca. 642,00 €
Irisimplantat Modell C0/F0 (Ophtec)	Nein	Ja	> 120	Silikon	ca. 1600,00 €
Irisimplantat Modell C1/F1 (Ophtec)	Ja	Ja	> 120	Silikon	ca. 1900,00 €

Die individuell angefertigte, flexible, undurchsichtige und zuschneidbare künstliche Iris besteht aus Silikon und bietet eine operative Behandlungsmöglichkeit gegen die Blendempfindlichkeit bei gleichzeitig vielversprechendem ästhetischem Ergebnis. Sie ist prinzipiell kunstlinsenunabhängig nutzbar. Die Vorderfläche wird mit verschiedenfarbigem Silikon individuell anhand eines Fotos der Restiris des betroffenen oder des Partnerauges modelliert. Die Rückfläche besteht aus einer glatten, schwarzen und lichtundurchlässigen Silikonschicht. Der Durchmesser beträgt 12,8 mm und beinhaltet einen fixen Pupillendurchmesser von 3,35 mm. Die Materialstärke nimmt vom Pupillarrand (0,4 mm) bis zum peripheren Rand (0,25 mm) ab. Das Implantat ist alternativ mit einem eingebetteten Gewebegitter erhältlich, um bei geplanter Nahtfixation eine Durchwanderung der Nähte aus dem weichen Silikon zu verhindern.

**Figure 3:**
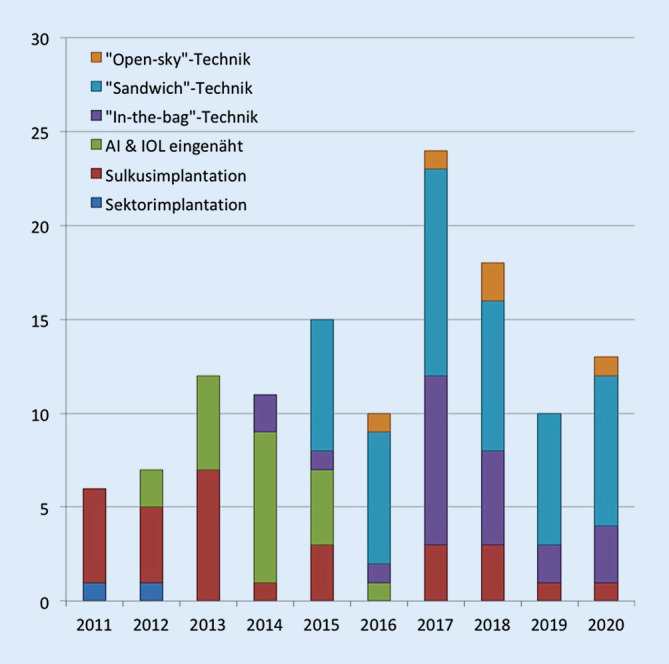

Insgesamt wurden durch den Erstautor von 2011 bis 2020 über 120 Irisimplantationen mit dieser Silikoniris bei verschiedensten Ausgangsbefunden durchgeführt (Abb. 3).

## Chirurgische Herangehensweise

### Grundlegende Vorbereitungen

Die Kosten des Eingriffs können im Rahmen des stationären Aufenthaltes einerseits über die DRG und die Kosten für das Implantat andererseits über das Zusatzentgelt ZE2015-108 abgedeckt werden. Bei BG-Fällen ergeben sich allgemein keine Abrechnungsprobleme.

Die Implantation der künstlichen Iris erfolgt über einen kornealen Zugang (ca. 2,8 mm) oder einen sklerokornealen Zugang (ca. 7 mm) in den Sulcus ciliaris oder den Kapselsack und nie in die Vorderkammer. In der Regel bleibt die Restiris des Patienten erhalten und wird als anteriore Begrenzung zum Kammerwinkel genutzt. Aufgrund des Risikos der Kataraktentwicklung wird die Implantation nur bei aphaken oder pseudophaken Augen empfohlen und durchgeführt.

Der erforderliche Durchmesser der künstlichen Iris wird individuell über den Weiß-zu-Weiß-Durchmesser gemessen. Alternativ ist dabei auch ein geringfügig kleinerer Durchmesser als der Durchmesser des Sulcus ciliaris (ca. 0,5 mm weniger) anzustreben, um einer mechanischen Irritation entgegenzuwirken. Praktischerweise entspricht dies etwa dem des Hornhautdurchmessers. Falls erforderlich, wird die künstliche Iris mit einem Einmaltrepan getrimmt. Die richtige Trepanationsgröße ist wichtig für die Zentrierung der Pupille nach der Operation. In den Fällen, in denen Nähte erforderlich sind, verwenden wir nichtresorbierbare 9‑0- oder 10-0-Polypropylen-Nähte (Ethicon).

**Figure 4:**
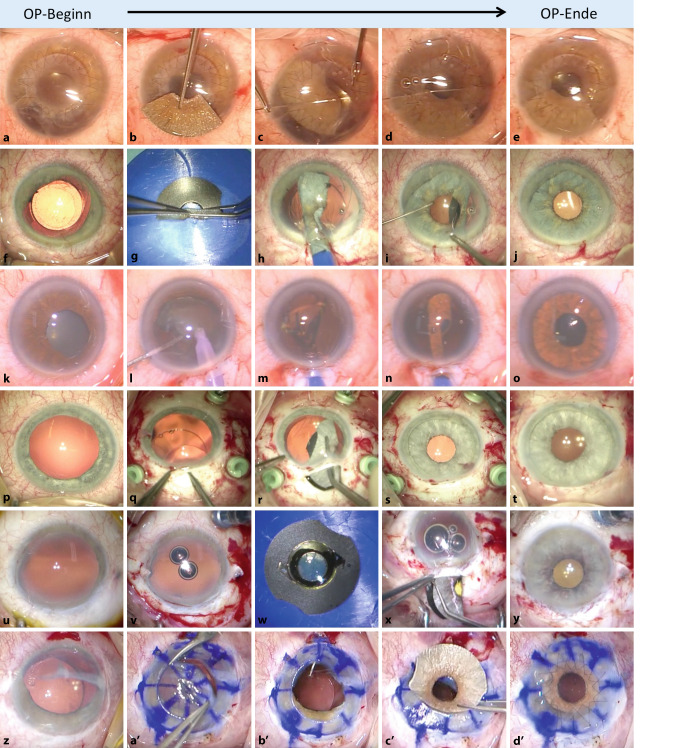

Bisher wurden verschiedene Implantationstechniken – z. T. in leichten Variationen – in Abhängigkeit von der Ausgangsituation beschrieben (Abb. 4; [3, 17, 19, 31, 36]):

1. **Sektorförmig ausgeschnittene künstliche Iris, Seite-an-Seite an der Restiris vernäht **(Abb. 4a–e)
  Neben der Implantation einer kompletten künstlichen Iris können auch nur deren Teilstücke implantiert werden. Bei kleineren, nicht direkt verschließbaren Irisdefekten (über bis 4 bis 5 Uhrzeiten) ist es möglich, lediglich ein Segment einer künstlichen Iris mit Gewebe zu implantieren, um einen sektorförmigen Irisdefekt zu überbrücken: Zunächst wird die künstliche Iris mit einer Schere auf die passende Größe zurechtgeschnitten. Die Implantation in die Vorderkammer selbst ist einfach, die folgenden notwendigen intrakameralen Irisnähte sind aber komplex und zeitaufwendig. Außerdem besteht die Gefahr der Nahtdurchwanderung an den Fixationspunkten aufgrund der noch intakten und mobilen Restiris. Die Implantation einer kompletten künstlichen Iris ist handwerklich einfacher durchzuführen und nicht nachteilig. Bei kleineren Defekten kann dennoch die sektorförmige Implantation einer dementsprechend zugeschnittenen Prothese in Erwägung gezogen werden. Der Vorteil besteht darin, dass der Sulcus ciliaris nicht zirkulär mit der Irisprothese „belegt“ ist und somit das Risiko für ein Sekundärglaukom möglicherweise reduziert wird.
2. **Injektorunterstützte Implantation einer kompletten künstlichen Iris in den Sulcus ciliaris bei pseudophaken Augen **(Abb. 4f–j)
  Diese Technik kann bei bereits pseudophaken Augen mit größeren Irisdefekten oder bei persistierender Mydriasis angewendet werden. Eine Implantation der künstlichen Iris ist aufgrund der hohen Flexibilität des Silikons durch Injektion mit einem Injektorsystem (Viscoject™-Bio 2.2 Injector Set) in Kleinschnitttechnik (ab 2,8 mm) möglich. Dieses Verfahren setzt eine stabile IOL im Kapselsack voraus. Dabei wird die aufgerollte künstliche Iris in den Sulcus ciliaris injiziert. Die Operationstechnik ist letztlich vergleichbar mit der Implantation einer additiven Intraokularlinse [1, 9, 11, 28, 29, 37, 39]. Sie ist einfach, schnell durchführbar und erfordert wie die nächste Technik keine Fixationsnähte.
3. **Injektorunterstützte Implantation einer kompletten künstlichen Iris und einer IOL in den Kapselsack **(Abb. 4k–o)
  Diese Technik eignet sich für Augen mit größeren Irisdefekten und kann in Kombination mit einer Kataraktoperation durchgeführt werden. Zunächst wird eine Standardkataraktextraktion durchgeführt. Der Durchmesser der Kapsulorhexis sollte etwas größer geplant werden (ca. 6 mm). Nach Implantation der Kunstlinse und einem Kapselspannring wird die künstliche Iris zusammen in den Kapselsack („in the bag“) implantiert. Die Bestimmung des adäquaten Durchmessers (ca. 9 mm) ist bei der kapselsackfixierten Variante allerdings schwieriger als bei der Sulcusfixation. Darüber hinaus kommt es während der Implantation in den Kapselsack zu einem nicht zu unterschätzenden Stress auf die Zonulafasern. Diese Operationstechnik bietet insgesamt ein recht geringes postoperatives Komplikationsspektrum und sollte daher bei gleichzeitig geplanter Kataraktoperation unbedingt in Erwägung gezogen werden.
4. **Komplette Irisprothesenimplantation in Kombination mit einer sklerafixierten Intraokularlinse bei Aniridie und Aphakie **(Abb. 4p–t)
  Bei großen Irisdefekten und fehlender Kapselunterstützung ist eine Fixierung von Linse und künstlicher Iris an der Sklera erforderlich. Zunächst wird eine für die Sulcusimplantation geeignete IOL über einen sklerokornealen Tunnel implantiert. Die IOL kann mit vorgelegten 10-0-Polypropylen-Fäden mit verschiedenen Nahttechniken an die Sklera geschützt unter Skleradeckeln fixiert werden. Die Linsenhaptiken werden an der Sklera in der 3‑ und 9‑Uhr-Position und die Irisnähte sinnvollerweise in der 6‑ und 12-Uhr-Position gelegt, um eine axiale Verkippungsneigung beider Implantate gegenseitig zu neutralisieren. Zu den Nachteilen dieser Technik gehören eine längere Eingriffsdauer, die Notwendigkeit für eine große Inzision (ca. 7 mm), einhergehend mit einem erhöhten Risiko für eine Hypotonie, 2 weitere Öffnungen in der Sklera an der 12- und 6‑Uhr-Position, die Gefahr einer Skleraldeckelerosion und spießender Fadenenden sowie eine erhöhte Infektionsgefahr aufgrund der Notwendigkeit von 2 zusätzlichen Skleranähten. Aus diesen Gründen erscheint die folgende Operationstechnik (Technik 5) vorteilhafter.
5. **Irisprothesenimplantation in Kombination mit einer individuellen IOL bei Aniridie und Aphakie **(Abb. 4u–y)
  Alternativ zum vorherigen Verfahren kann eine beliebige handelsübliche faltbare einteilige Kunstlinse auf die Rückseite der Silikoniris genäht und dann als „Doppelprothese“ gefaltet in das Auge implantiert werden [15, 33]. Um das „Sandwich-Implantat“ möglichst kompakt zu halten, können die nicht benötigten Haptiken der IOL abgetrennt werden. Die Nahtfixation erfolgt intraskleral an der 3‑ und 9‑Uhr-Position der künstlichen Iris und an der Sklera. Es ist wichtig, die Befestigungspunkte genau in gegenüberliegender Position vorzulegen, um eine gute Pupillenzentrierung zu erreichen. Das „Paket“ mit den vorgelegten Fäden kann dann mit einer Pinzette gefaltet sklerokorneal eingeführt werden. Weitere Vorteile dieser Technik sind eine kleinere Inzision durch ein faltbares Iris-Linsen-Paket, die Notwendigkeit von nur 2 statt 4 skleralen Befestigungspunkten und eine konsekutiv kürzere Operationsdauer. Unserer Meinung nach ist diese Technik die beste Wahl für die Rekonstruktion von Augen mit großen Irisdefekten und Aphakie.
6. **„Open-sky“-Implantation im Rahmen einer perforierenden Keratoplastik **(Abb. 4z–d’)
  Sowohl bei pseudophaken, aphaken oder phaken Augen mit Irisdefekten und gleichzeitig bestehenden Hornhautnarben bietet sich diese kombinierte Operationstechnik an [14]. Dabei wird die künstliche Iris auf den erforderlichen Durchmesser trepaniert, ggf. eine IOL aufgenäht bzw. eine „Open-sky“-Kataraktextraktion durchgeführt und die Iris durch die Hornhautöffnung in den Sulcus ciliaris implantiert und die Öffnung abschließend mit dem Hornhauttransplantat verschlossen. Der Vorteil dieser Technik ist die Möglichkeit, die Implantation der künstlichen Iris (sowie IOL) und die Keratoplastik in einer einzigen Sitzung durchzuführen, wodurch ein zusätzliches operatives Trauma vermieden wird. Dies ist insbesondere für die Schonung des Endothels des Hornhauttransplantats von Bedeutung.

## Postoperative Nachsorge

**Figure 5:**
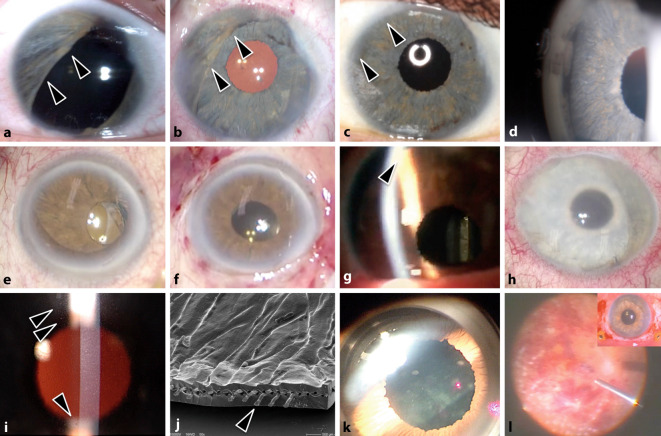

Die postoperative Nachsorge der Patienten sollte neben den üblichen Untersuchungen regelmäßige Augeninnendruckkontrollen und Endothelzellzahlmessungen beinhalten. Der Augenhintergrund behandelter Augen kann trotz fehlender Möglichkeit zur Pupillenerweiterung angesichts einer ausreichenden Öffnungsgröße von 3,35 mm fundoskopisch untersucht werden, evtl. auch mittels Indentation. Eine Vitrektomie oder auch Kapsulotomie mit dem Nd:YAG-Laser ist ebenfalls ohne wesentliche Einschränkungen durch die neue, starre Pupille möglich (Abb. 5k, l).

## Funktionelle Ergebnisse

Die bisherigen Ergebnisse sind vielversprechend [25, 26, 32]. In einer prospektiven Studie zur Untersuchung der funktionellen Ergebnisse und der Patientenzufriedenheit nach chirurgischer Irisrekonstruktion wurden 32 konsekutive Patienten mit traumatischen Irisdefekten untersucht [23]. Hinsichtlich der bestkorrigierten Sehschärfe und des Augeninnendrucks zeigten sich keine statistisch signifikanten Unterschiede beim Vergleich vor und nach der Implantation. Es wurde eine erwartete signifikante Reduktion um ca. 79 % der Pupillenöffnung festgestellt. Die Kontrastsensitivität stieg statistisch signifikant um ca. 11 % an (Pelli-Robson-Chart), während die Endothelzellzahl nach der Operation statistisch signifikant um ca. 5 % abnahm. Hinsichtlich der Vorderkammertiefe und des Kammerwinkels zeigten sich keine statistisch signifikanten Unterschiede. Die subjektive Beeinträchtigung durch Blendung und Kosmetik verbesserte sich deutlich und statistisch signifikant. Die Patientenzufriedenheit mit dem Gesamtergebnis betrug 8,9 ± 1,5 von bestmöglichen 10 Punkten.

Die kombinierte Implantation einer monofokalen Intraokularlinse und einer künstlichen Iris wird inzwischen routinemäßig zur Behandlung von Patienten mit Aphakie und Aniridie durchgeführt [15, 33]. Es wurde bereits in vitro an der optischen Bank (OptiSpheric IOL Pro II, Trioptics, Wedel, Deutschland) gezeigt, dass ein Annähen der IOL an die AI die optische Qualität nicht in einem relevanten Ausmaß reduziert [16].

Die Verwendung der präoperativen Biometrie (durchgeführt in üblicher Weise) ergab eine gut vorhersagbare postoperative Refraktion bei 39 untersuchten Augen. Es ist kein Korrekturfaktor erforderlich, gleich welche AI-Operationstechnik mit gleichzeitiger IOL-Implantation erfolgt [15].

Eine weitere klinische Untersuchung konzentrierte sich auf die funktionellen Ergebnisse nach einer ebensolchen kombinierten Iris- und IOL-Implantation bei Aniridiepatienten [20]. In dieser Beobachtungsstudie wurden 59 aniride und aphake Augen mit einer „Doppelprothese“ behandelt. Der monokulare bestkorrigierte Visus verbesserte sich signifikant von 0,7 logMAR auf 0,3 logMAR.

## Ästhetische Ergebnisse

In einer prospektiven klinischen Studie mit Analyse der prä- und postoperativen Fotos von 70 Patienten wurden die subjektiven Bewertungen der 70 behandelten Patienten sowie von 50 Augenärzten und 30 Laien auf Fragebögen sowie objektiv mittels Bildbearbeitungssoftware ausgewertet [38]. Generell zeigten sich subjektiv und objektiv gute Ergebnisse hinsichtlich der Farbauswahl. Die gelieferte AI war im Durchschnitt eher etwas heller als die Restiris und die des Partnerauges. Eine geringe Pupillendezentrierung korrelierte stark mit einem statistisch signifikant besseren ästhetischen Ergebnis. Die Pupillenzentrierung war ein Schlüsselfaktor, der signifikant mit dem Ausmaß der ästhetischen Zufriedenheit korrelierte. Die recht geringe Pupillenasymmetrie bzw. Anisokorie wurde nie bemängelt oder als störend empfunden.

## Komplikationen

Aufgrund der meist vielfach vorgeschädigten Augen besteht ein nicht zu vernachlässigendes, breit gefächertes intra- und postoperatives Komplikationsrisiko unterschiedlichen Schweregrades [2, 5]. Dazu gehören moderate Dezentrierungen und Subluxationen der künstlichen Iris aus der optischen Achse (Abb. 5e–g), kurz- und langfristige Anstiege des Augeninnendruckes, eine Abnahme der Endothelzellzahl, chronische Reizzustände mit Makulaödem und Hornhautdekompensation (Abb. 5h). Die Implantation einer künstlichen Irisprothese kann zu einem Restirisretraktionssyndrom führen (Abb. 5a–d; [22]). Farbveränderungen wurden an der Restiris nicht beobachtet.

Patienten mit kongenitaler Aniridie haben neben der Aniridie ein deutliches Glaukomrisiko, das durch die Implantation einer Irisprothese in den Sulcus ciliaris noch potenziert werden kann. Daher ist bei kongenitaler Aniridie eine Implantation der Irisprothese in den Kapselsack im Rahmen einer Kataraktoperation zu bevorzugen (s. oben beschriebene Technik 3 und Abb. 4k–o).

In einer Studie traten bei 25,5 % unerwartete Ereignisse in unterschiedlichem Ausmaß auf (Tab. 2; [21]). Die Komplikationsrate sank beim selben Chirurgen von 83,3 % im ersten Jahr auf 13,3 % im vierten Jahr. Die signifikante Reduktion der Komplikationen nach 12 Implantationen impliziert, dass das Verfahren bei geringen Operationszahlen nicht zu empfehlen ist. Prinzipiell ist die künstliche Iris wieder explantierbar.

**Table 2:** 

Gradeinteilung		Beschreibung	*n*	%
0 (keine)	Regelrechter postoperativer Verlauf	–	38	67,1
1 (mild)	*Vorübergehende *unerwartete Ereignisse ohne chirurgische Intervention	Rezidivierende Blutungen mit IOD-Anstieg	1	2,0
		Geringe stabile Dezentrierung der Iris (≥ 1 mm)	1	2,0
		Kapsulotomie bei Nachstar	2	3,9
2 (moderat)	Dauerhafte unerwartete Ereignisse mit *konservativer *Intervention	Fadendurchwanderung an der Kunstiris	1	2,0
		Neu aufgetretenes Glaukom	3	5,9
		Neu aufgetretene Hornhautdekompensation	3	5,9
3 (schwer)	Dauerhafte unerwartete Ereignisse, mit *chirurgischer *Intervention	Fadenlockerung der Fixationsnähte	2	3,9
		Sub‑/Dislokation der Kunstiris	3	5,9
		Neu aufgetretene Glaskörperstränge oder Synechien	2	3,9
		Neu aufgetretenes Glaukom (Ahmed-Valve-Implantat)	2	3,9
		Neu aufgetretene Hornhautdekompensation (Amnionmembran)	2	3,9
		Neu aufgetretene Hornhautdekompensation (perforierende Keratoplastik)	5	9,8
		CMÖ	3	5,9
		Netzhautablösung	1	2,0

**Figure 6:**
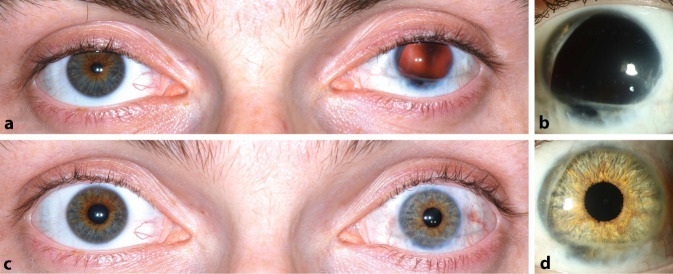

Zusammenfassend zeigt sich bei der Implantation der künstlichen Iris eine relativ flache Lernkurve. Die Komplikationsrate liegt bei ca. einem Drittel aller Patienten, was aber v. a. auch der Vorgeschichte der schwer traumatisierten Augen geschuldet ist. Daher ist es empfehlenswert, den Eingriff an einem Zentrum durchzuführen, an dem auch die Möglichkeit besteht, evtl. Folgeprobleme zu behandeln. Insgesamt ist die Implantation der künstlichen Iris trotz der bekannten Risiken eine weitere gute Therapieoption zur Behandlung eines traumatisch bedingten Irisverlustes. Neben guten funktionellen und gleichzeitig ansprechenden kosmetischen Ergebnissen kann eine hohe Patientenzufriedenheit erreicht werden (Abb. 6).

## Fazit für die Praxis

- Es steht eine Vielzahl von Implantationstechniken zur Verfügung. Die am besten geeignete Vorgehensweise ist anhand der Ausgangssituation zu wählen.
- Die Implantation der künstlichen Iris erfolgt nie in die Vorderkammer, sondern immer in den Sulcus ciliaris oder den Kapselsack. Bei phakem Auge kommt sie nicht zum Einsatz.
- Die Wahl des richtigen Durchmessers der künstlichen Iris entscheidet mit über die gute Zentrierung und damit letztlich das ästhetische Ergebnis.
- Die Anwendung des Irisimplantates ist auch in der Hand eines Chirurgen mit „Pole-to-pole“-Erfahrung eine Herausforderung.
- Die postoperative Nachsorge beinhaltet Kontrollen insbesondere hinsichtlich des Augeninnendruckes, der Endothelzellzahl und (chronischer) Reizzustände.
